# Frecuencia de positividad de la prueba de intradermorreacción a tuberculina en una cohorte de pacientes con artritis reumatoide

**DOI:** 10.7705/biomedica.5416

**Published:** 2021-09-22

**Authors:** Wilson Bautista-Molano, Liza González, Daniel Fernández-Ávila, Rosa Cardozo, Óscar Ruiz

**Affiliations:** 1 Organización Sanitas Internacional/Colsanitas, Bogotá, D.C., Colombia Organización Sanitas Internacional/Colsanitas BogotáD.C Colombia; 2 Facultad de Medicina, Universidad Militar Nueva Granada, Bogotá, D.C., Colombia Universidad Militar Nueva Granada Facultad de Medicina Universidad Militar Nueva Granada BogotáD.C Colombia; 3 Facultad de Medicina, Universidad El Bosque, Bogotá, D.C., Colombia Universidad El Bosque Facultad de Medicina Universidad El Bosque BogotáD.C Colombia; 4 Facultad de Medicina, Fundación Universitaria Sanitas, Bogotá, D.C., Colombia Facultad de Medicina Fundación Universitaria Sanitas BogotáD.C Colombia; 5 Departamento de Medicina Interna, Hospital Universitario San Ignacio, Pontificia Universidad Javeriana, Bogotá, D.C., Colombia Pontificia Universidad Javeriana Departamento de Medicina Interna Pontificia Universidad Javeriana BogotáD.C Colombia

**Keywords:** artritis reumatoide/epidemiología, tuberculosis, riesgo, terapia biológica, Arthritis, rheumatoid/epidemiology, tuberculosis, risk, biological therapy

## Abstract

**Introducción.:**

La artritis reumatoide es una enfermedad autoinmunitaria, crónica y deformante asociada con discapacidad. Quienes la padecen reciben inmunosupresores y tienen un gran riesgo de desarrollar tuberculosis. La prueba de intradermorreacción a la tuberculina se utiliza como tamización en quienes van a recibir terapia biológica.

**Objetivo.:**

Evaluar la frecuencia de positividad en la prueba de intradermorreacción a la tuberculina en una cohorte de pacientes con artritis reumatoide.

**Materiales y métodos.:**

Se hizo un estudio descriptivo de corte transversal de una cohorte de pacientes con artritis reumatoide a quienes se les practicó la prueba de tuberculina antes de iniciar la terapia biológica o en el momento del cambio de tratamiento. Los pacientes presentaban enfermedad moderada o grave y eran candidatos para iniciar o cambiar de terapia biológica. Se definió el valor de ≥6 mm como punto de corte para la positividad de la prueba y se hizo un análisis descriptivo de cada una de las variables.

**Resultados.:**

Se incluyeron 261 pacientes con artritis reumatoide, 92 % de ellos eran mujeres, la edad promedio fue de 55 años (desviación estándar, DE=13,92) y el tiempo desde el diagnóstico era de 12,3 años (DE=8,54). La frecuencia de positividad de la prueba fue de 15,71 % (n=41). Nueve de los 41 pacientes positivos habían recibido la prueba previamente (entre 1 y 6 años antes), todos con resultado negativo; 18 (43,9 %) de ellos venían recibiendo tratamiento con glucocorticoides y todos los 41 (100 %) recibían metotrexate.

**Conclusiones.:**

La frecuencia de positividad de la prueba de tuberculina en pacientes colombianos con artritis reumatoide fue de aproximadamente 16 %. Se recomienda optimizar las estrategias para detectar esta condición y darle un tratamiento oportuno y, así, disminuir el riesgo de reactivación de la tuberculosis.

## Abstract (en)

**Introduction:**

Rheumatoid arthritis is an autoimmune, chronic, and deforming condition associated with disability. Patients are immunosuppressed and at high risk of developing tuberculosis. The tuberculin skin test is used to screen candidates for biological therapy.

**Objective:**

To evaluate the frequency of positivity of the tuberculin skin test in a cohort of Colombian patients with rheumatoid arthritis.

**Materials and methods:**

We conducted a descriptive cross-sectional study including patients with rheumatoid arthritis receiving the tuberculin skin test prior to the start or at the time of the change of biological therapy. The patients' condition was moderate or severe and they were candidates for initiation or change of biological therapy. We defined the value of ≥6 mm as the cut-off point for a positive tuberculin skin test and performed a descriptive analysis for each of the variables considered.

**Results:**

In total, 261 patients with rheumatoid arthritis were included, 92 % of whom were women; the average age was 55 years (SD=13.92) and the time from diagnosis, 12.3 years (SD=8.54). The frequency of positive tuberculin skin tests was 15.71% (n=41). Of the 41 positive patients, nine had previously had the test (1 to 6 years before), all of them with negative results; 18 of these were receiving glucocorticoids (43.9%) and all of them (100%) were being treated with methotrexate.

**Conclusions:**

The frequency of positivity of the tuberculin skin test in these Colombian patients diagnosed with rheumatoid arthritis was around 16%. We reco0mmend optimizing strategies aimed at an optimal detection of this condition and the timely initiation of treatment to reduce the risk of tuberculosis reactivation.

La artritis reumatoide es una enfermedad sistémica de etiología autoinmunitaria que se caracteriza por poliartritis simétrica episódica, crónica, erosiva y deformante, de impacto en salud pública y que se asocia con discapacidad a mediano y largo plazo ^1^^,^^2^. Durante la última década, la terapia biológica, incluido el bloqueo farmacológico del factor de necrosis tumoral *(Tumor Necrosis Factor,* TNF), se ha convertido en una estrategia terapéutica eficaz para el manejo de enfermedades inflamatorias crónicas como esta ^3^ y, por lo tanto, el esquema de tratamiento se incluye en guías y recomendaciones internacionales ^4^.

La tuberculosis es una enfermedad granulomatosa causada por la infección con *Mycobacterium tuberculosis.* El bacilo responsable se transmite en las gotas de saliva de pacientes infectados a personas sanas, lo que desencadena una reacción a nivel alveolar caracterizada por una reacción de macrófagos y de células dendríticas que intentan fagocitar y destruir el bacilo. Algunas de estas células permanecen en el pulmón y otras migran a los ganglios linfáticos, donde se produce la activación de linfocitos T que migran a los granulomas en formación ^5^. El granuloma previene la actividad del bacilo de la tuberculosis y algunos pacientes pueden depurar la carga de bacilos; sin embargo, otros se mantienen en un estado de latencia y pueden reactivarse, incluso décadas después de la primoinfección. La reactivación depende del estado inmunológico del huésped. La tuberculosis activa se desarrolla en 5 a 15 % de los pacientes contagiados con el bacilo, y un gran porcentaje de este grupo son personas inmunocomprometidas ^6^.

Se considera que cerca de dos billones de personas, aproximadamente un tercio de la población mundial, pueden tener tuberculosis latente, cifra que ha venido en aumento con la infección por el virus de inmunodeficiencia humana (HIV) ^7^. Por otra parte, se estima que se diagnostican ocho millones de casos de tuberculosis al año en el mundo, y entre 2 y 3 millones de personas mueren a causa de la enfermedad ^8^. En el caso específico de Colombia, la tuberculosis es una de las principales enfermedades de gran impacto en salud pública. En el 2002, cerca de dos terceras partes de las entidades territoriales del país presentaban incidencias por encima del promedio nacional (26 casos por cada 100.000 habitantes) ^9^, con un incremento en la tasa de incidencia de casos bacteriológicamente confirmados entre el 2012 y el 2018 ^10^.

La gran mayoría de quienes entran en contacto con el bacilo nunca desarrollan la infección porque su sistema inmunológico funciona adecuadamente. Una de las citocinas más importantes de la reacción inmunológica contra el bacilo es el TNF, una molécula crítica para la integridad del granuloma ^11^, por lo que los individuos expuestos o que van a ser expuestos a antagonistas farmacológicos del TNF se encuentran en gran riesgo de desarrollar tuberculosis: una vez iniciado el tratamiento, el riesgo relativo de enfermar se incrementa entre 1,6 y 25,1 veces dependiendo de la situación clínica particular de cada paciente ^12^.

Entre los métodos paraclínicos de detección de casos de tuberculosis, se encuentran la prueba de intradermorreacción a la tuberculina y la de QuantiFERON™. La primera, denominada aquí como prueba de tuberculina *(Purifbd Protein Derivative,* PPD), es poco específica en pacientes que han recibido la vacuna BCG (bacilo de Calmette Guérin) y poco sensible en aquellos que están en estado de inmunosupresión ^13^. La prueba de QuantiFERON™ y la T-SPOT TB™ miden la reacción de los linfocitos T *in vitro* frente a los antígenos que no están presentes en la vacuna BCG e, incluso, en varias de las cepas de *Mycobacterium* no tuberculosas, por lo que constituyen pruebas más específicas que la de tuberculina ^14^; no obstante, con ellas se ha reportado un mayor número de casos de conversión de pruebas negativas a falsos positivos y de positivas a falsos negativos, que con la de tuberculina ^15^.

La prueba de tuberculina es la típica prueba de sensibilidad retardada, cuya célula efectora es el macrófago mononuclear. Tiene varias etapas: la de reconocimiento mediada por células T CD4 y CD8 de antígenos extraños en las células presentadoras de antígeno; la fase de activación en que las células T proliferan y liberan citocinas proinflamatorias; la fase efectora en que las células inflamatorias migran al tejido que contiene el antígeno, y la fase de resolución en que los macrófagos eliminan el antígeno ^16^. Es por este mecanismo fisiopatológico que su lectura debe interpretarse teniendo en cuenta los siguientes factores: integridad del sistema inmunitario, presencia o no de *M. tuberculosis,* factores socioeconómicos y epidemiológicos del huésped, características clínicas y radiológicas del paciente y, por último, verificación de la presencia de una "reactivación" o "reinfección" ^17^.

A partir de estas precisiones, se han establecido varios puntos de corte útiles para la interpretación de la prueba: la reacción cutánea negativa (induración menor de 5 mm) que se interpreta como un resultado negativo en pacientes adultos que no han tenido contacto con casos de tuberculosis activa en los tres meses anteriores y no tienen factores de riesgo por inmunosupresión; la reacción cutánea intermedia (induración de 5 a 10 mm) que se interpreta como prueba positiva en personas con factores de riesgo alto para tuberculosis, por ejemplo, aquellos con infección por HIV, en quienes han tenido contacto con pacientes con tuberculosis activa, tienen evidencia radiológica de lesiones previas de tuberculosis, o han recibido trasplantes o medicamentos inmunosupresores como glucocorticoides en dosis de más de 15 mg durante más de tres meses; y, la reacción cutánea mayor (induración mayor de 10 mm) que se interpreta como prueba positiva en pacientes con induración entre 5 y 9 mm pero con condiciones médicas que incrementan el riesgo de tuberculosis (diabetes mellitus y enfermedades hematológicas o reticuloendoteliales como leucemia o enfermedad de Hodgkin), usuarios de drogas endovenosas o con factores de riesgo para adquirir el HIV, aquellos con carcinoma de cabeza, cuello y pulmón, enfermedad renal terminal, pérdida de peso mayor de 10 % en los seis meses anteriores, y gastrectomía o derivación yeyuno-ileal ^18^.

En el ejercicio médico, la prueba de tuberculina es la que con mayor frecuencia se usa para la tamización de pacientes con enfermedades reumatológicas que van a recibir terapias biológicas, entre ellas, inhibidores del TNF. Se considera que los pacientes con artritis reumatoide tienen inmunosupresión porque la enfermedad altera el normal funcionamiento del sistema inmunológico y reciben tratamiento con glucocorticoides que, asociados con otros medicamentos, entre ellos el metotrexato, amplifican ese efecto depresor en el sistema inmunológico.

En Colombia, son escasos los estudios en que se evalúa la prevalencia de positividad de la prueba de tuberculina en pacientes con enfermedades inflamatorias crónicas, y es urgente determinarla en nuestro contexto clínico. Por ello, se propuso como objetivo de este estudio evaluar la frecuencia de positividad de la prueba de tuberculina en una cohorte de seguimiento de pacientes con diagnóstico de artritis reumatoide antes del inicio de la terapia biológica o de la modificación del tratamiento.

## Materiales y métodos

Se hizo un estudio descriptivo de corte transversal en el que se documentaron y analizaron los datos de un grupo de pacientes de una cohorte en seguimiento clínico en el programa de artritis reumatoide. Se seleccionó a aquellos que ya habían recibido la prueba de tuberculina según el registro en la historia clínica antes del inicio de la terapia biológica o en el momento del cambio.

La muestra fue no probabilística, por conveniencia y secuencial, a partir de la cohorte de los individuos que asistían regularmente a la consulta de reumatología. Se definió el valor de 6 o más milímetros como punto de corte para considerar positiva la prueba de tuberculina, puesto que se trataba de pacientes que en el momento de la prueba venían recibiendo tratamiento inmunomodulador con antirreumáticos modificadores de la enfermedad o con terapia biológica, y cuya enfermedad era moderada a grave, por lo que en ese momento eran candidatos para el inicio o el cambio de terapia biológica. A todos los pacientes se les tomaron, además, radiografías de tórax y se les hizo el examen físico para detectar adenopatías cervicales, axilares o inguinales como parte de la tamización que rutinariamente se hace para tuberculosis latente.

Los datos sobre los pacientes provenían de la historia clínica electrónica de seguimiento en la consulta de reumatología. Se diseñó una base de datos para recopilar la información consolidada cada cuatro semanas para detectar datos erróneos o faltantes y garantizar un adecuado control de calidad. El programa usado fue Access, que permite eliminar los datos repetidos y hacer las validaciones de los valores de los registros. Se hizo el análisis descriptivo de cada una de las variables de estudio, y se utilizaron medidas de tendencia central y variabilidad para describir las características de la población. En cuanto al manejo estadístico de los datos, se utilizó el programa Stata™, versión 12.

### Consideraciones éticas

Por tratarse de un estudio descriptivo sin intervención ni modificación de las variables biológicas, fisiológicas, psicológicas o sociales de los pacientes, la investigación se consideró de bajo riesgo y no fue necesario el consentimiento informado por escrito. Los datos se mantuvieron en absoluta confidencialidad en cumplimiento de los principios establecidos en el Código de Núremberg, la Convención de Helsinki (1964) y la Resolución 008430 de 1993 del Ministerio de Salud y Protección Social de la República de Colombia. El almacenamiento de la información en la base de datos se ajustó a lo previsto en la Ley 1582 de 2012 y solo los investigadores principales tuvieron acceso a la base de datos.

## Resultados

**Figura 1:**
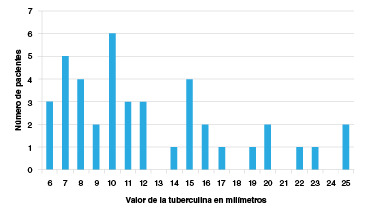
Valor de la tuberculina (PPD) en milímetros (mm) en el grupo de pacientes con tuberculosis latente

Se analizó la información de 261 pacientes, 91,9 % de ellos eran mujeres (n=240), la edad promedio fue de 55,06 años (DE=13,92) y el tiempo promedio desde el diagnóstico de artritis reumatoide fue de 12,28 años (DE=8,54 años). La frecuencia de positividad de la prueba de intradermorreacción a tuberculina para este grupo fue de 15,71 % (n=41). El resultado de la prueba medido en milímetros para los casos positivos fue variable, con un rango entre los 6 y 25 milímetros (figura 1).

**Figura 2:**
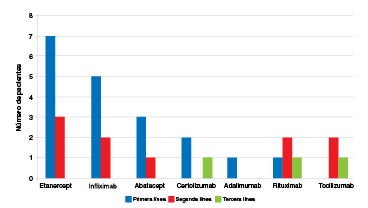
Número de pacientes en quienes se detectó tuberculosis latente por uso previo de terapia biológica y por uso de tratamientos de primera, segunda o tercera línea

Del total de pacientes con resultado negativo (n=199), la prueba ya se había aplicado a 41 (entre 1 y 5 años antes) y, en todos ellos, había sido negativa. De los 41 pacientes con resultado positivo en la prueba de estudio, nueve ya habían sido sometidos a esta en el pasado (entre 1 a 6 años antes), con resultado negativo en todos ellos. En el momento de la prueba de tuberculina, 19 pacientes del grupo de aquellos con tuberculosis latente habían recibido, por lo menos, un producto biológico previamente y estaban a la espera del cambio de la terapia biológica; los 22 casos restantes correspondían a pacientes que venían en tratamiento con antirreumáticos modificadores de la enfermedad y la prueba de tuberculina se solicitaba para iniciar el tratamiento biológico. De los 19 pacientes que habían recibido biológicos previamente, 10 habían recibido dos y tres habían recibido tres (figura 2). En cuanto al uso de glucocorticoides, se encontró que, de los 41 pacientes con tuberculosis latente, 18 (43,9 %) venían recibiendo glucocorticoides (prednisona en dosis de 10 a 40 mg día) y todos ellos (100 %) recibían tratamiento modificador de la enfermedad con metotrexato.

En cuanto a la radiografía de tórax, se encontraron 10 casos con hallazgos anormales en el grupo de los pacientes con tuberculina negativa y ningún caso en el grupo de aquellos con tuberculosis latente. Los pacientes con alteraciones en la radiografía de tórax fueron remitidos a neumología, pero no se detectaron casos de tuberculosis pulmonar. Los pacientes con prueba de tuberculina positiva recibieron un tratamiento profiláctico con 300 mg de isoniacida al día durante nueve meses, con suplemento de piridoxina, y continúan en seguimiento clínico en el programa. En el examen físico, ninguno de los pacientes con prueba de tuberculina positiva presentó adenopatías cervicales, axilares o inguinales.

## Discusión

El principal objetivo del presente estudio fue evaluar la frecuencia de resultados positivos en la prueba de tuberculina en una cohorte de seguimiento de 261 pacientes con diagnóstico de artritis reumatoide. En este grupo de pacientes, la prueba fue solicitada antes del inicio o cambio de tratamiento con terapia biológica, siguiendo las recomendaciones de gestión de riesgo para la prescripción de este tipo de tratamiento ^19^. Se encontró que la frecuencia estimada de positividad de la prueba de tuberculina para este grupo fue de cerca del 16 % y en ninguno de los casos hubo hallazgos clínicos o radiográficos de relevancia en el tórax.

La artritis reumatoide se ha asociado con un incremento del riesgo de infecciones debido a su fisiopatología y al uso de tratamientos inmunosupresores. La reactivación de la infección latente de tuberculosis es un problema grave en estos pacientes, especialmente después de recibir terapia anti-TNF. La inhibición del TNF impide la formación del granuloma tuberculoso y fomenta el crecimiento de las micobacterias. Después de una infección de tuberculosis, la recomendación es suspender el tratamiento con inhibidores de TNF hasta que la infección se haya resuelto, a pesar de su efecto favorable para suprimir la inflamación ^20^^,^^21^.

La prueba de tuberculina detecta la intradermorreacción a antígenos micobacterianos producto de la precipitación proteica a partir de la filtración del cultivo de *M. tuberculosis* (PPD). Se la considera la prueba de referencia para el diagnóstico de tuberculosis latente al detectar la hipersensibilidad por exposición previa al bacilo. Es positiva cuando la induración es mayor de 5 mm en personas con mayor riesgo de tuberculosis activa por estar en tratamiento con inmunosupresores en dosis equivalentes a 15 mg por día de prednisona o más o con antagonistas del TNFα ^22^. La gran frecuencia de positividad de la tuberculina encontrada en el presente estudio refleja lo esperado en un país endémico para tuberculosis. Según datos del Instituto Nacional de Salud, durante el 2014 se reportaron 10.849 casos nuevos de tuberculosis en todas sus formas, es decir, una incidencia de 23,02 casos por 100.000 habitantes ^23^. Esta cifra se ha incrementado en la última década, con un reporte de 14.446 casos en el 2018, para una incidencia de 26,9 casos por 100.000 habitantes ^10^.

La gran mayoría de individuos expuestos a *M. tuberculosis* desarrolla la forma latente, en la que la infección es contenida en granulomas a partir de los cuales puede reactivarse la enfermedad. En el presente estudio, se encontró que el 44 % de los pacientes con tuberculosis latente venía recibiendo glucocorticoides (prednisona en dosis de 10 a 40 mg día) y el 100 % recibía tratamiento modificador de la enfermedad con metotrexato. Esta circunstancia se relaciona con un incremento del riesgo de este tipo de infección y constituye una variable que debe considerarse en pacientes que reciben dichos tratamientos, dada la gran probabilidad de reactivación de la tuberculosis. En ese sentido, es claro que la detección de tuberculosis latente antes de iniciar la terapia biológica puede minimizar complicaciones posteriores. En línea con lo anterior, en varios estudios se han evaluado biomarcadores adicionales y otros procedimientos para la detección temprana de la infección ^24^.

La frecuencia reportada de la prueba de tuberculina en este estudio es concordante con estudios previos realizados en otros países latinoamericanos. Se reportó una frecuencia del 12,4 % en pacientes argentinos con artritis reumatoide sometidos a la prueba de tuberculina, asociada con la administración de altas dosis de glucocorticoides ^25^. En un estudio chileno en 41 pacientes con esta condición, se reportó una positividad de la prueba mayor de 5 mm en el 36 % de los participantes (Gil G, Neira O, Marcone P, Sabagh E. Tuberculine reactivity in Chilean RA patients. Abstract XlVth Congress of the Pan-American League of Associations for Rheumatology: CATEGORY: Epidemiology. J Clin Rheumatol. 2006;12(Suppl 4): S8). Según otro estudio en Latinoamérica, que incluyó pacientes colombianos tratados por otra enfermedad inflamatoria crónica, la espondiloartritis, la prevalencia de tuberculosis activa fue del 3,3 % (IC_95%_ 1,8-5,7), significativamente mayor que la de la población general (0,32 %). El riesgo relativo estandarizado para esta población fue de 10,3 comparado con la población general ^26^. Hasta donde se pudo comprobar, este es uno de los primeros estudios en que se determinó la frecuencia de positividad de la prueba de tuberculina en un grupo significativo de pacientes colombianos con artritis reumatoide en seguimiento clínico.

El estudio tuvo varias limitaciones. En primer lugar, debido a diferencias entre los países, los resultados sobre la reactividad de la prueba no pueden ser extrapolados (ni siquiera a otros países de la región) y, por lo tanto, deben interpretarse en este contexto.

En segundo lugar, el efecto *booster* no pudo verificarse en todos los pacientes del estudio. La prueba de dos pasos y reacción reforzada para determinarlo consiste en la administración de una segunda prueba entre una y cinco semanas después de una primera prueba falsamente negativa. La prueba se hace en algunos pacientes en quienes la capacidad para reaccionar al antígeno de la tuberculina disminuye con el tiempo, lo que da lugar a una reacción falsamente negativa (por ejemplo, cuando la infección tuberculosa es muy antigua) ^27^. Sin embargo, la utilidad de esta prueba ha sido cuestionada en otros estudios ^28^.

En tercer lugar, la prueba confirmatoria de QuantiFERON TB™ (considerada la de referencia) no se utilizó en los pacientes que fueron reactivos en la prueba de tuberculina. La solicitud de esta prueba confirmatoria no es habitual en la práctica clínica de los reumatólogos colombianos, especialmente porque no está incluida en el plan básico de atención en salud por su alto costo.

En cuarto lugar, no se recolectó la información relacionada con el estrato socioeconómico y las condiciones de hacinamiento de la vivienda de los pacientes de la cohorte, aspectos estos que se han establecido como factores de riesgo para el desarrollo de tuberculosis y podrían ofrecer un contexto adicional desde el punto de vista socioeconómico para estratificar el riesgo de estos pacientes. Dado que no se tuvo la información previa de la prueba de tuberculina, no es posible hablar de porcentaje de pacientes con viraje en el resultado, ya que solo se registraron los datos de 9 pacientes del grupo de positivos y 41 del de negativos.

Aunque no se incluyeron otras variables útiles para estratificar el riesgo (tratamientos farmacológicos previos, resultado de prueba de tuberculina previa, estrato socioeconómico, hacinamiento y contacto epidemiológico), estos resultados reflejan una realidad objetiva del impacto de la tuberculosis latente en pacientes con artritis reumatoide y constituyen la línea de base para el desarrollo de estudios posteriores que permitan estratificar el riesgo de desarrollar tuberculosis en pacientes candidatos a terapia biológica y, por consiguiente, minimizar el impacto de esta infección en un país endémico como el nuestro. Asimismo, los estudios futuros permitirían determinar el rendimiento operativo de la prueba de tuberculina *Vs.* el QuantiFERON™ en pacientes con enfermedades inflamatorias crónicas, con el fin de diseñar e implementar algoritmos y diagramas de flujo para la adopción de decisiones en nuestro contexto clínico.

Por último, los resultados aportan información relevante sobre el comportamiento de la tuberculosis en una cohorte de pacientes con artritis reumatoide y permitirá el diseño, implementación y optimización de procesos de atención, lo que hoy se diiculta por la escasa información disponible. En conclusión, la gran frecuencia de positividad de la prueba de tuberculina en estos pacientes reclama estrategias que optimicen la detección de dicha condición y el inicio oportuno del tratamiento para disminuir el riesgo de reactivación de la tuberculosis.
